# Characterization of amoxicillin and clavulanic-acid-responsive CD4+ And CD8+ T-cells in patients with co-amoxiclav-induced liver injury

**DOI:** 10.1186/2045-7022-4-S3-P42

**Published:** 2014-07-18

**Authors:** Seung-Hyun Kim, Katy Saide, Ye Yuan, Ann Daly, Ana Alfirevic, Munir Pirmohamed, Kevin Park, Dean Naisbitt

**Affiliations:** 1 Ajou University School of Medicine, Dept. of Allergy and Clinical Immunology, South Korea; 2 University of Liverpool, MRC Centre for Drug Safety Science, UK; 3 Newcastle University, Institute of Cellular Medicine, UK; 4 University of Liverpool, Department of Molecular and Clinical Pharmacology, UK

## Background

Co-amoxiclav, a combination antibiotic consisting of amoxicillin and clavulanic acid, is one of the most commonly prescribed anti-microbial drugs. Unfortunately, human exposure is associated with liver injury and in rare cases, acute liver failure has been reported. The risk of liver injury appears be higher when amoxicillin/clavulanic acid is used in combination. Little is known about the mechanistic basis of co-amoxiclav-induced liver injury. However, several HLA class I and II genotypes have recently been shown to affect susceptibility, which indicates a potential role for the adaptive immune system. Thus, the objective of this study was to (1) investigate whether drug responsive T-cells are detectable in the peripheral blood of patients with co-amoxiclav-induced liver injury, (2) define antigen specificity and (3) characterize the phenotype and function of antigen-specific T-cells.

## Method

The lymphocyte transformation test and IFN ELIspot were used to detect drug responsive T-cells in the peripheral blood of 7 patients with liver injury. T-cells were then cloned to measure (1) proliferative responses in the presence of amoxicillin and clavulanic acid, (2) cell surface marker expression by flow cytometry and (3) the profile of secreted cytokines/cytolytic molecules by ELIspot.

## Results

Lymphocytes from 5 out of 7 patients with co-amoxiclav-induced liver injury were found to proliferate and/or secrete IFN when cultured with amoxicillin or clavulanic acid. Amoxicillin (n=78; CD4+ and CD8+) and clavulanic acid (n=5; all CD4+) -responsive T-cell clones were generated by serial dilution. Activation of the clones was drug-specific. Proliferative responses and IFN, IL13, FasL, granzyme B and perforin secretion was detected with amoxicillin or clavulanic acid, but not with the alternative antibiotic. Detailed structure activity studies using the amoxicillin clones and 5 additional antibiotics identified T-cell responses against ampicillin alone.

## Conclusion

These studies have characterized T-cells in the peripheral blood of patients with co-amoxiclav-induced liver injury that are selectively activated with either amoxicillin or clavulanic acid. These data indicate that the adaptive immune system participates in the disease pathogenesis. Further work is needed to elucidate why amoxicillin, when combined with clavulanic acid, is a common cause of liver injury.

